# High sensitivity and enhanced antibiotic stewardship of the BioFire Joint Infection Panel in acute, but not chronic, prosthetic joint infection of the knee

**DOI:** 10.1128/spectrum.00286-25

**Published:** 2025-06-18

**Authors:** Tsung-Li Lin, Chen-Wei Yeh, Chun-Hao Tsai, Mao-Wang Ho, Hsiu-Hsien Lin, Po-Ren Hsueh

**Affiliations:** 1 Department of Orthopedics, China Medical University Hospital 38020, Taichung, Taiwan; 2 Department of Sports Medicine, College of Health Care, China Medical University 684961, Taichung, Taiwan; 3 Division of Infectious Diseases, Department of Internal Medicine, China Medical University Hospital, China Medical University 38020, Taichung, Taiwan; 4 Department of Internal Medicine, China Medical University Hospital, School of Medicine, China Medical University 665159 , Taichung, Taiwan; 5 Department of Laboratory Medicine, China Medical University Hospital, China Medical University 38020, Taichung, Taiwan; 6 PhD Program for Aging, School of Medicine, China Medical University 665159 , Taichung, Taiwan; NHLS Tygerberg/Stellenbosch University, Cape Town, Western Cape, South Africa

**Keywords:** Joint Infection Panel, prosthetic joint infection, synovial fluid culture, antibiotic exposure, antibiotic stewardship

## Abstract

The BioFire Joint Infection (JI) Panel is a multiplex polymerase chain reaction assay developed for rapid pathogen detection in synovial fluid, particularly for suspected prosthetic joint infections (PJI). However, its diagnostic sensitivity varies by clinical context and pathogen, and the impact of prior antibiotic exposure on sample quality and PJI stewardship remains unclear. This prospective study evaluated the diagnostic performance of the Investigational Use Only JI Panel versus conventional synovial fluid culture, using Musculoskeletal Infection Society criteria and symptom onset within 3 weeks to define acute PJI. Fifty-four fresh synovial fluid samples from patients with suspected knee PJI were analyzed. In acute PJI, the JI Panel demonstrated comparable sensitivity to synovial fluid culture (80% vs. 95%; *P* = 0.096), while significantly reducing time to pathogen identification (mean 18.2 vs. 84.6 hours, *P* < 0.001), enabling earlier targeted antibiotic therapy in 83.3% of cases. Overall sensitivity across all PJI cases was moderate (50%) and significantly lower in chronic PJI (16.7%), reflecting limited panel coverage and reduced detection in prolonged infections. Prior antibiotic exposure reduced sensitivity for both methods, though not significant (*P* = 0.127). The absence of key pathogens such as *Staphylococcus epidermidis* and *Cutibacterium acnes* further limited diagnostic yield in chronic cases. The JI Panel offers a rapid and clinically impactful tool for diagnosing acute PJI and guiding antibiotic stewardship. However, its limitations in chronic PJI and susceptibility to antibiotic pretreatment restrict its standalone diagnostic utility. Therefore, it should complement, not replace, traditional culture methods, particularly in chronic PJI cases.

**IMPORTANCE:**

The JI Panel demonstrates high sensitivity for acute PJI but lower sensitivity for chronic infections. The ability of the JI Panel to rapidly identify pathogens in acute cases plays a significant role in improving antibiotic stewardship, ensuring timely and appropriate treatment. Given the lower sensitivity for chronic PJI, further research could focus on improving the detection of pathogens that are commonly involved in chronic infections. While the JI Panel is a promising tool for acute PJI diagnosis and supports rapid antibiotic stewardship, its limitations in chronic cases and under antibiotic exposure must be addressed to maximize its clinical utility.

## INTRODUCTION

Knee arthroplasty is a highly effective procedure for alleviating the symptoms of advanced knee osteoarthritis. However, prosthetic joint infection (PJI) remains the most common cause of failure following knee arthroplasty, affecting 1%–2% of patients undergoing primary procedures (1). The economic burden and impact on patients’ quality of life are significant, making PJI a serious concern (2). From the orthopedic surgeon’s perspective, the management of PJI depends on its classification as either acute or chronic, with distinct treatment strategies for each. Acute PJI is typically treated with debridement, antibiotics, and implant retention (DAIR), while chronic PJI often requires the removal of the prosthesis for effective management (3). Accurate and timely diagnosis is critical in treating PJI; however, no single diagnostic test offers a reliable, high-accuracy method for confirming the infection (4).

The diagnostic criteria for PJI include the presence of two positive cultures or a sinus tract. In addition, supplementary tools such as synovial fluid analysis and serum biomarkers assist in the evaluation, as outlined by the 2018 Musculoskeletal Infection Society (MSIS) criteria (5). Accurate diagnosis of PJI requires clinicians to integrate clinical findings, imaging results, and a range of laboratory tests (6). Culture-based diagnostics remain the gold standard, but approximately 5%–42% of PJI cases show negative results for pathogen (7). Factors that contribute to false-negative results include prior antibiotic use before sampling, the presence of fastidious pathogens, and issues like prolonged storage and transportation delays, all of which can compromise the quality of the sample and hinder pathogen growth (8).

With advancements in molecular biology techniques, the rapid detection of pathogens associated with infectious diseases has become increasingly feasible. These innovations allow for more accurate and timely identification of causative agents, improving diagnostic precision and facilitating prompt, targeted treatment (9–11). The U.S. Food and Drug Administration-approved BioFire Joint Infection (JI) Panel, a multiplex polymerase chain reaction (PCR) test, can detect 31 pathogens (15 Gram-positive bacteria, 14 Gram-negative bacteria, 2 yeasts) and 8 antimicrobial resistance (AMR) gene targets within 1 hour (12). Several studies have demonstrated its promising results, showing improved sensitivity and reduced wait times compared to traditional culture methods (13–15). However, most studies have combined native joint infection (NJI) and PJI cases, which limits the specific applicability of the findings to PJI alone (12, 14–20). Only a few studies have focused exclusively on PJI (13, 21). One key finding is the relatively low overall sensitivity of the JI Panel due to its inability to detect certain pathogens, such as *Staphylococcus epidermidis* (*S. epidermidis*) and *Cutibacterium acnes* (*C. acnes*), particularly in the setting of early acute PJI (13, 18). Furthermore, the optimal timing for applying the JI Panel, the impact of prior antibiotic exposure on sample quality, and its role in antibiotic stewardship for PJI remain unclear.

This prospective study was designed to compare the diagnostic performance of the JI Panel to traditional synovial fluid culture in patients with suspected PJI of the knee. The study hypothesized that there would be no significant differences in the diagnostic accuracy of the JI Panel for detecting acute and chronic PJI of the knee. In addition, the study aimed to evaluate the impact of antibiotic exposure within two weeks prior to sampling and analyze the time required to initiate targeted antibiotic therapy.

## MATERIALS AND METHODS

### Synovial fluid sample collection

The inclusion criteria included adult patients (>20 years) who had undergone primary total knee arthroplasty (TKA) and with suspected PJI. All total knee prostheses were implanted using a cemented technique. The femoral and tibial components were composed of cobalt-chromium alloys, whereas the tibial insert and patellar component were fabricated from ultra-high-molecular-weight polyethylene. Inclusion was irrespective of prior use of parenteral or oral antibiotics; however, this was the first surgical intervention for suspected PJI in these patients. Exclusion criteria included patients with confirmed PJI who had already undergone surgery, who developed PJI after revision TKA for aseptic failure, and who had recurrent PJI following a two-stage exchange procedure. Fresh synovial fluid specimens were obtained preoperatively through knee arthrocentesis performed by a single senior orthopedic surgeon between February and September 2024. The specimens were immediately sent to the central laboratory for testing without prior storage. PJI diagnosis was based on the 2018 criteria established by the MSIS (5). The clinical records noted whether antibiotics were administered within 2 weeks before sample collection. This prospective study was approved by the Research Ethics Committee (approval number: CMUH112-REC3-190). All participants provided informed consent prior to enrollment.

### JI Panel methodology

This study employed an Investigational Use Only (IUO) version of the JI Panel, which is functionally identical to the commercially available *in vitro* diagnostic version. The testing process adhered to the manufacturer’s protocol, as outlined below: 0.2 mL of synovial fluid was used per test, and the sample was mixed with the manufacturer’s provided sample buffer and hydration solution. The prepared mixture was added to the pouch of the JI Panel. The system incubated the sample for 1 hour. Then the BioFire instrument automatically performed the following steps: nucleic acid extraction, reverse transcription, and nucleic acid amplification. Finally, the BioFire instrument analyzed the amplified nucleic acids and displayed the test results directly on the JI Panel interface.

### Synovial fluid culture

Following the allocation of 0.2 mL of synovial fluid for the JI Panel testing, the remaining sample was used for a comprehensive synovial fluid examination. The distribution and culture protocols included as follows: (i) synovial fluid analysis: routine analysis for cell count, differential, crystal examination, and leukocyte esterase, (ii) blood culture bottles (Becton, Dickinson and Company, Sparks, MD, USA): synovial fluid was inoculated into aerobic and anaerobic blood culture bottles to enhance pathogen recovery, (iii) agar plate cultures: direct plating on aerobic and anaerobic agar plates for pathogen isolation, (iv) fungal cultures: specific pus fungal cultures to detect fungal pathogens, and (v) acid-fast stain and tuberculosis culture: screening for mycobacterial infections using acid-fast staining and culture on tuberculosis-specific media. We performed tuberculosis cultures by inoculating synovial fluid samples onto Löwenstein–Jensen medium and into the BACTEC MGIT 960 system (Becton, Dickinson and Company). Cultures were incubated at 37°C and monitored for up to 8 weeks. Mycobacterial species were identified using nucleic acid amplification tests or mass spectrometry. All aerobic and anaerobic cultures were incubated for a 14-day period to ensure adequate time for the detection of slow-growing organisms (22).

### Microbiological analysis

The results from intraoperative tissue cultures served as the reference standard for microbiological assessment. The analysis included the following. Pathogen-specific findings from the JI Panel were compared to those from synovial fluid culture in culture-positive PJI cases. Agreement rates and discrepancies between the two methods were recorded. Moreover, the distribution of detected pathogens was analyzed separately for overall, acute, and chronic PJI cases of the knee. Findings were categorized by infection type to highlight any variations in pathogen detection rates between acute and chronic cases. For consistency and clarity, pathogens rather than patients were used as the unit of analysis to represent collected microbiological data.

### Post-sampling treatment protocol

To classify PJI of the knee as acute or chronic, we used a 3-week symptom duration from onset as the cutoff. Surgical interventions and antibiotic strategies were tailored accordingly (23, 24). Surgical management for acute PJI was treated with DAIR, while chronic PJI was treated with a two-stage exchange procedure. We cultured five intraoperative periprosthetic tissue samples obtained during surgery to detect pathogens. The choice of antibiotics (empiric or targeted) depended on the timing and results of pathogen identification, either through the JI Panel or synovial fluid culture or intraoperative tissue culture. Clinical scenarios included the following: (i) pathogen identified by JI Panel: empiric antibiotics were shifted to targeted therapy immediately after the JI Panel identified the pathogen (within hours). If culture-based results, available after at least 72 hours, matched the JI Panel findings, the targeted antibiotics were continued; (ii) negative JI Panel results: empirical antibiotics were initiated. If culture results later revealed a pathogen, treatment was de-escalated to culture-based targeted antibiotics; (iii) negative results from both JI Panel and culture: empirical antibiotics were continued throughout the treatment course; and (iv) discrepancy between JI Panel and culture results: the definitive targeted therapy was determined collaboratively by the orthopedic surgeon, infection specialist, and microbiologists.

### Outcome measurement

The time to initiation of targeted antibiotics was extracted from medical records and analyzed to assess the impact of rapid pathogen identification on clinical decision-making and treatment timelines.

### Statistical analysis

Data were analyzed using Statistical Package for the Social Sciences (SPSS) software version 24.0 (SPSS Inc. Armonk, NY). Sensitivity was calculated for pathogens targeted by the JI Panel (on-JI Panel) and for synovial fluid culture; all pathogens detected through intraoperative tissue culture served as the reference standard and were expressed as a percentage of true-positive results among culture-positive cases. McNemar’s test was used to compare the diagnostic performance of the JI Panel with that of synovial fluid culture for detecting PJI. Wilson’s method was applied to estimate 95% confidence intervals for sensitivity and other proportions. Statistical significance was determined by a *P* value ≤ 0.05.

## RESULTS

### Demographics

**Fig 1:**
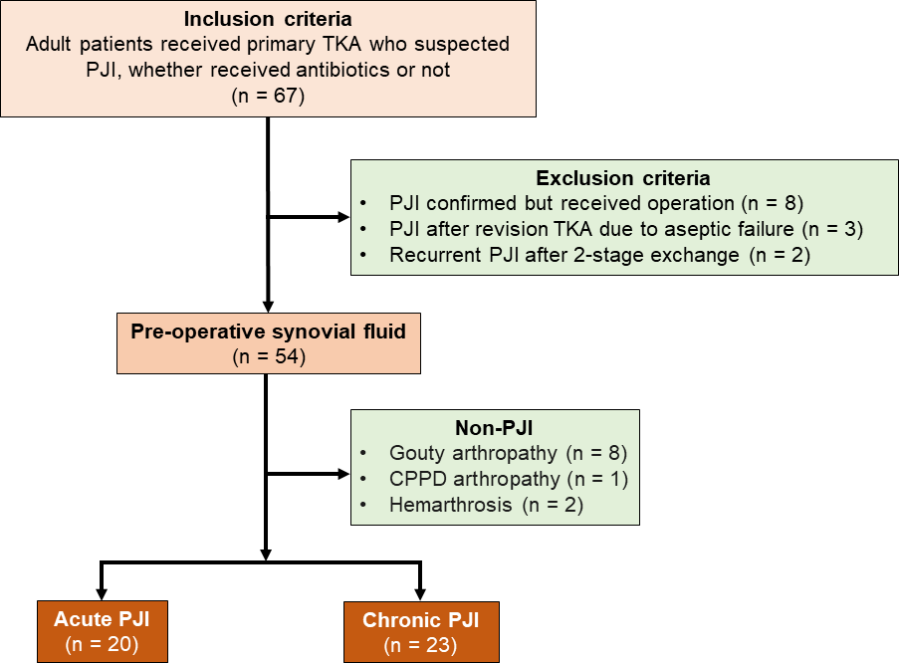
A Strengthening the Reporting of Observational Studies in Epidemiology flowchart outlining the study design. TKA, total knee arthroplasty; PJI, prosthetic joint infection; CPPD, calcium pyrophosphate dihydrate.

A total of 54 patients were included, with 11 non-PJI, 20 acute PJI, and 23 chronic PJI. The overall PJI rate was 79.6% (43/54), with the following breakdown: culture-positive rate 79.1% (34/43), culture-negative rate 20.9% (9/43), and polymicrobial infection rate 11.8% (4/34). A Strengthening the Reporting of Observational Studies in Epidemiology (STROBE) flowchart outlining the study design is provided in Fig. 1. In the culture-positive PJI group, the breakdown of pathogen detection by the JI Panel and synovial fluid culture is demonstrated in Table 1, with on-JI Panel pathogens (detected by JI Panel): 57.9% (22/38), and off-JI Panel pathogens (detected by synovial fluid culture but not included in the JI Panel) 42.1% (16/38).

**TABLE 1:** Pathogen-specific findings of the BioFire JI Panel and synovial fluid cultures in prosthetic joint infection (PJI) of the knee

	No. of samples with pathogens detected by indicated method/total no. of samples with pathogen detected by both methods	
Pathogen (*n* = 38)	JI Panel	Synovial fluid culture
On-JI Panel		
*Staphylococcus aureus*	12/13	12/13
*Streptococcus agalactiae*	3/3	3/3
*Streptococcus species*	1/1	1/1
*Enterococcus faecalis*	0/1	1/1
*Serratia marcescens*	1/1	1/1
*Pseudomonas aeruginosa*	1/1	1/1
*Klebsiella pneumoniae*	0/1	1/1
*Escherichia coli*	1/1	1/1
Off-JI Panel		
*Staphylococcus epidermidis*	0/9	9/9
*Streptococcus oralis*	0/2	2/2
*Staphylococcus capitis*	0/1	1/1
*Staphylococcus caprae*	0/1	1/1
*Streptococcus anginosus*	0/1	1/1
*Cutibacterium acnes*	0/1	1/1
*Candida glabrata*	0/1	1/1

### Pathogen distribution

**Fig 2:**
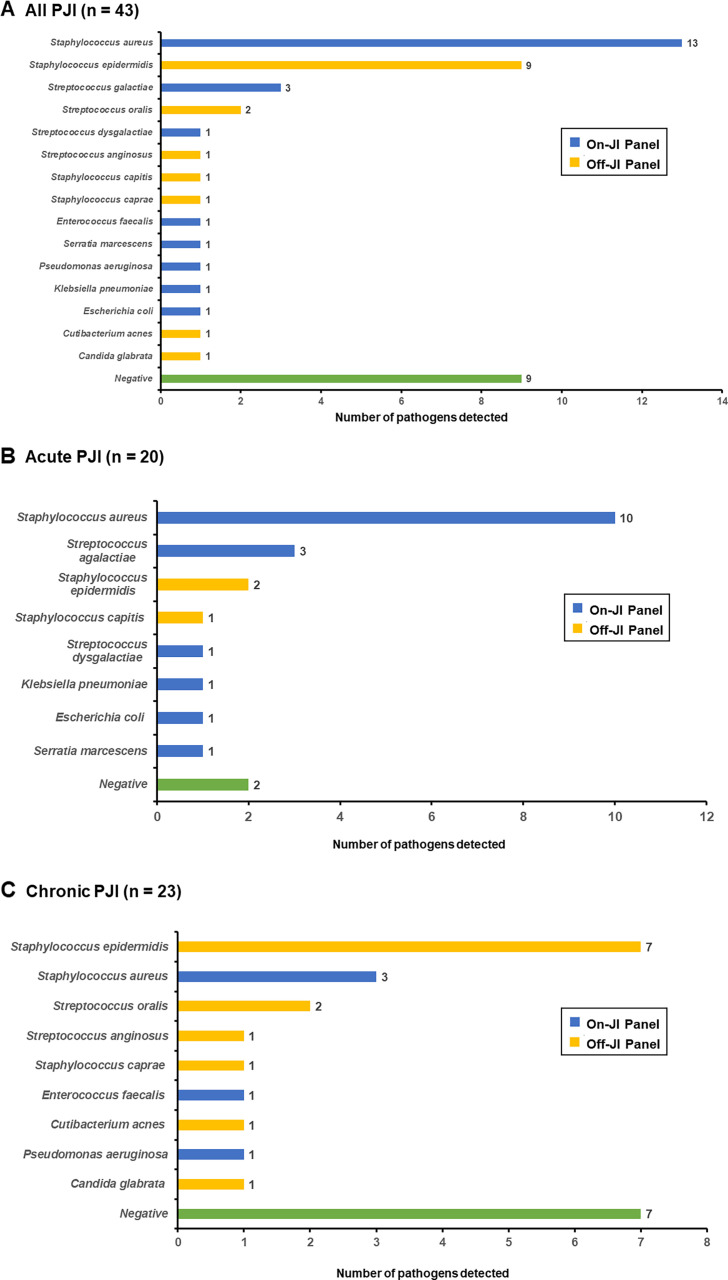
Number of detected pathogens on- and off-JI Panel among (**A**) all PJI, (**B**) acute PJI, and (**C**) chronic PJI.

The distribution of detected pathogens among all, acute, and chronic PJI cases is detailed in Table 2. In all PJI (Fig. 2A), *Staphylococcus* spp. was the predominant pathogen, comprising the top three pathogens as follows: *Staphylococcus aureus* (34.2%), *S. epidermidis* (23.7%), and *Streptococcus agalactiae* (7.9%). In acute PJI (Fig. 2B), *Staphylococcus* spp. continued to be the most common pathogen. The top three pathogens detected were as follows: *S. aureus* (50%), *S. agalactiae* (15%), and *S. epidermidis* (10%). In chronic PJI (Fig. 2C), *Staphylococcus* spp. dominated as in acute PJI, but with a different distribution of species: *S. epidermidis* (38.9%), *S. aureus* (16.7%), and *Streptococcus oralis* (11.1%).

**TABLE 2:** Distribution of detected pathogens by BioFire JI Panel and synovial fluid cultures among all, acute, and chronic PJI

	No. (% of pathogen)		
Pathogen	All PJI (*n* = 38)	Acute PJI (*n* = 20)	Chronic PJI (*n* = 18)
*Staphylococcus aureus*	13 (34.2)	10 (50.0)	3 (16.7)
*Staphylococcus epidermidis*	9 (23.7)	2 (10.0)	7 (38.9)
*Streptococcus agalactiae*	3 (7.9)	3 (15.0)	0 (0.0)
*Streptococcus oralis*	2 (4.7)	0 (0.0)	2 (11.1)
*Streptococcus dysgalactiae*	1 (2.6)	1 (5.0)	0 (0.0)
*Streptococcus anginosus*	1 (2.6)	0 (0.0)	1 (5.6)
*Staphylococcus capitis*	1 (2.6)	1 (5.0)	0 (0.0)
*Staphylococcus caprae*	1 (2.6)	0 (0.0)	1 (5.6)
*Enterococcus faecalis*	1 (2.6)	0 (0.0)	1 (5.6)
*Serratia marcescens*	1 (2.6)	1 (5.0)	0 (0.0)
*Pseudomonas aeruginosa*	1 (2.6)	0 (0.0)	1 (5.6)
*Klebsiella pneumoniae*	1 (2.6)	1 (5.0)	0 (0.0)
*Escherichia coli*	1 (2.6)	1 (5.0)	0 (0.0)
*Cutibacterium acnes*	1 (2.6)	0 (0.0)	1 (5.6)
*Candida glabrata*	1 (2.6)	0 (0.0)	1 (5.6)
Negative	9 (23.7)	2 (10.0)	7 (38.9)

### Diagnostic performance of the JI Panel

The performances of the JI Panel and synovial fluid culture were analyzed using intraoperative tissue cultures as the standard reference. The sensitivity of the JI Panel for diagnosing acute PJI was 80.0%, which was comparable to the sensitivity of synovial fluid culture (95.0%) (*P* = 0.096). For all PJI (including acute and chronic cases), the sensitivity of the JI Panel was 50.0%, significantly lower than the sensitivity of synovial fluid culture. For chronic PJI, the sensitivity of the JI Panel was notably low at 16.7%, while synovial fluid culture had a much higher sensitivity (Table 3).

**TABLE 3:** Diagnostic accuracy of JI Panel, synovial fluid culture, and intraoperative tissue culture in patients with suspected PJI of the knee^*a*^

Diagnostic performance	JI Panel	Synovial fluid culture	Intraoperative tissue culture	*P* value
Specificity, 95% CI	90.9% (10/11), 71–99	100% (11/11), 93–100	Reference standard	0.318
All PJI, sensitivity, 95% CI	50.0% (19/38), 41–68	97.4% (37/38), 82–100	Reference standard	**0.001**
Acute PJI, sensitivity, 95% CI	80.0% (16/20), 68–92	95.0% (19/20), 81–99	Reference standard	0.096
Chronic PJI, sensitivity, 95% CI	16.7% (3/18), 5–39	100% (18/18), 97–100	Reference standard	**<0.001**

^*a*^ CI, confidence interval. Bold font: *P* < 0.05.

### Sensitivity of on-JI Panel pathogens

For all PJI and acute PJI, the sensitivity of the on-JI Panel was comparable to that of synovial fluid culture, though still showing reduced performance in chronic PJI (with sensitivity at 60%), as detailed in Table 4.

**TABLE 4:** Diagnostic accuracy of on-JI Panel, synovial fluid culture, and intraoperative tissue culture in patients with PJI of the knee^*a*^

Diagnostic accuracy	On-JI Panel	Synovial fluid culture	Intraoperative tissue culture	*P* value
All PJI, sensitivity, 95% CI	86.4% (19/22), 67–93	97.4% (37/38), 82–100	Reference standard	0.105
Acute PJI, sensitivity, 95% CI	94.1% (16/17), 73–100	95.0% (19/20), 81–99	Reference standard	0.834
Chronic PJI, sensitivity, 95% CI	60.0% (3/5), 44–71	100% (18/18), 97–100	Reference standard	**0.001**
Antibiotics within 2 weeks before sampling, sensitivity, 95% CI	85.7% (12/14), 65–92	95.8% (23/24), 70–99	Reference standard	0.127

^*a*^ CI, confidence interval. Bold font: *P* < 0.05.

### Impact of prior antibiotic exposure

A total of 72.1% (31/43) of PJIs had prior antibiotic exposure within 2 weeks before sampling. Both the on-JI Panel and synovial fluid culture showed reduced sensitivity when prior antibiotic exposure occurred, but the differences in sensitivity between the two methods were not statistically significant (*P* = 0.127). The on-JI Panel sensitivity was 85.7% with prior antibiotic exposure, while the sensitivity of synovial fluid culture was 95.8% (Table 4).

### Antibiotic stewardship

The time to start targeted antibiotics was significantly faster in patients whose pathogens were identified by the JI Panel (18.2 hours, range: 2.5–96.5 hours). Of these patients, 83.3% (15/18) were diagnosed with acute PJI. For patients whose pathogens were identified only through synovial fluid culture, the time to start targeted antibiotics was 84.6 hours (range: 65–182 hours) (*P* < 0.001). The on-JI Panel significantly enhanced antibiotic stewardship, especially in acute PJI cases, by reducing the empiric antibiotic course by 66.4 hours. Table 5 provides detailed data for each suspected PJI patient, including antibiotics usage within two weeks before sampling, JI Panel and synovial fluid and intraoperative tissue culture test results, surgical procedure, and time to targeted antibiotics.

**TABLE 5:** Detailed data of each suspected PJI patient^*a*^

Case no.	Antibiotic use within 2 weeks before sampling	JI Panel	Synovial fluid culture	Surgical procedure	Intraoperative tissue culture (positive/total samples)	Time to targeted antibiotics used (hours)
Acute PJI						
1	No	MSSA	MSSA	DAIR	MSSA (3/5)	3
2	No	MSSA	MSSA	DAIR	MSSA (5/5)	4.5
3	No	MSSA	MSSA	DAIR	MSSA (4/5)	3
4	No	MSSA	MSSA	DAIR	MSSA (5/5)	3
5	Yes	MSSA	MSSA	DAIR	MSSA (2/5)	4.5
6	Yes	MSSA	MSSA	DAIR	MSSA (2/5)	5
7	Yes	MSSA	MSSA	DAIR	MSSA (3/5)	3.5
8	Yes	MSSA, *Escherichia coli*	*E. coli*	DAIR	MSSA (1/5), *E. coli* (2/5)	5
9	Yes	MSSA	MSSA, carbapenem-resistant *Klebsiella pneumoniae*	DAIR	MSSA (2/5), carbapenem-resistant *Klebsiella pneumoniae* (2/5)	96.5
10	No	MRSA	MRSA	DAIR	MRSA (5/5)	2.5
11	No	*Streptococcus agalactiae*	*S. agalactiae*	DAIR	*S. agalactiae* (5/5)	3
12	Yes	*S. agalactiae*	*S. agalactiae*	DAIR	*S. agalactiae* (2/5)	5
13	Yes	*S. agalactiae*	*S. agalactiae*	DAIR	*S. agalactiae* (3/5)	3.5
14	Yes	*Streptococcus* spp.	*Streptococcus dysgalactiae*	DAIR	*S. dysgalactiae* (4/5)	4.5
15	No	*Serratia marcescens*	*S. marcescens*	DAIR	*S. marcescens* (5/5)	3
16	No	Negative	MRSE	DAIR	MRSE (4/5)	84
17	Yes	Negative	MRSE	DAIR	MRSE (2/5)	75.5
18	Yes	Negative	Methicillin-susceptible *S. capitis*	DAIR	Methicillin-susceptible *S. capitis* (3/5)	78
19	Yes	Negative	Negative	DAIR	Negative	Empiric
20	Yes	Negative	Negative	DAIR	Negative	Empiric
Chronic PJI						
21	Yes	MSSA	MSSA	2-stage	MSSA (2/5)	4
22	Yes	MSSA	MSSA, MRSA	2-stage	MSSA (3/5), MRSA (1/5)	79.5
23	Yes	*Pseudomonas aeruginosa*	*P. aeruginosa*, *Enterococcus faecalis*	2-stage	*P. aeruginosa* (2/5), *E. faecalis* (2/5)	94
24	Yes	Negative	MRSE	2-stage	MRSE (1/5)	82.5
25	No	Negative	MRSE	2-stage	MRSE (2/5)	72
26	No	Negative	MRSE	2-stage	MRSE (3/5)	70.5
27	Yes	Negative	MRSE	2-stage	MRSE (3/5)	78
28	Yes	Negative	MRSE	2-stage	MRSE (2/5)	68
29	Yes	Negative	MSSE	2-stage	MSSE (1/5)	65
30	Yes	Negative	Methicillin-resistant *S. caprae*	2-stage	Methicillin-resistant *S. caprae* (3/5)	74
31	No	Negative	MSSA, *Streptococcus oralis*	2-stage	MSSA (4/5), *S. oralis* (1/5)	75.5
32	No	Negative	*S. oralis*	2-stage	*S. oralis* (3/5)	74.5
33	Yes	Negative	*Streptococcus anginosus*	2-stage	*S. anginosus* (4/5)	96
34	Yes	Negative	MSSE	2-stage	MSSE (2/5)	76
35	Yes	Negative	*Cutibacterium acnes*	2-stage	*C. acnes* (2/5)	170
36	Yes	Negative	*Candida glabrata*	2-stage	*Candida glabrata* (5/5)	182
37	Yes	Negative	Negative	2-stage	Negative	Empiric
38	Yes	Negative	Negative	2-stage	Negative	Empiric
39	Yes	Negative	Negative	2-stage	Negative	Empiric
40	Yes	Negative	Negative	2-stage	Negative	Empiric
41	Yes	Negative	Negative	2-stage	Negative	Empiric
42	Yes	Negative	Negative	2-stage	Negative	Empiric
43	Yes	Negative	Negative	2-stage	Negative	Empiric
Non-PJI						
44	No	MRSA	Negative	–	–	–
45	No	Negative	Negative	–	–	–
46	No	Negative	Negative	–	–	–
47	No	Negative	Negative	–	–	–
48	No	Negative	Negative	–	–	–
49	No	Negative	Negative	–	–	–
50	No	Negative	Negative	–	–	–
51	Yes	Negative	Negative	–	–	–
52	Yes	Negative	Negative	–	–	–
53	Yes	Negative	Negative	–	–	–
54	Yes	Negative	Negative	–	–	–

^*a*^ DAIR, debridement, antibiotics, and implant retention; MSSA, methicillin-susceptible *Staphylococcus aureus*; MRSA, methicillin-resistant *Staphylococcus aureus*; MSSE, methicillin-susceptible *Staphylococcus epidermidis*; MRSE, methicillin-resistant *Staphylococcus epidermidis*; –, the procedure was not performed.

### Discrepancy analysis

A total of six discrepancy results were observed between the JI Panel and synovial fluid culture, as summarized in Table 6 and categorized as follows.

**TABLE 6:** Discrepancy results between the JI Panel, synovial fluid culture, and intraoperative tissue culture^*a*^

Case No.	JI panel	Synovial fluid culture	Intraoperative tissue culture (positive/total samples)	CRP (mg/dL)	ESR (mm/1 h)	Synovial fluid		
						WBC (μL)	PMN (%)	LE
Acute PJI								
8	MSSA, *Escherichia coli*	*E. coli*	MSSA (1/5), *E. coli* (2/5)	20.98	67	39,500	93	3+
9	MSSA	MSSA, *Carbapenem-resistant Klebsiella pneumoniae*	MSSA (2/5), *Carbapenem-resistant Klebsiella pneumoniae* (2/5)	13.61	80	30,300	90	3+
Chronic PJI								
22	MSSA	MSSA, MRSA	MSSA (3/5), MRSA (1/5)	2.34	102	8,963	86	2+
23	*Pseudomonas aeruginosa*	*P. aeruginosa, Enterococcus faecalis*	*P. aeruginosa* (2/5), *E. faecalis* (2/5)	3.82	114	10,235	81	2+
31	Negative	MSSA, *Streptococcus oralis*	MSSA (4/5), *S. oralis* (1/5)	3.09	94	25,430	93	3+
Non-PJI								
44	MRSA	Negative		1.03	8	550	3	-

^*a*^ CRP, C-reactive protein; ESR, erythrocyte sedimentation rate; LE, leukocyte esterase; MSSA, methicillin-susceptible *Staphylococcus aureus*; MRSA, methicillin-resistant *Staphylococcus aureus*.

(A) JI Panel false negative.

1. AMR gene false negative in cases 9 and 22.

The JI Panel failed to detect carbapenem-resistant *Klebsiella pneumoniae* (case 9) and methicillin-resistant *S. aureus* (MRSA) (case 22).

These false-negative results in detecting critical AMR genes led to a delay in the accurate identification of the pathogens. After multidisciplinary discussions, polymicrobial PJI was diagnosed, and targeted antibiotics were subsequently prescribed.

2*. Enterococcus faecalis* false negative in case 23.

In a polymicrobial PJI, the JI Panel failed to detect *E. faecalis* and identified only *Pseudomonas aeruginosa*. As a result, targeted antibiotics were prescribed for both pathogens, but the failure to detect *E. faecalis* could have impacted the initial management if not for the clinical recognition of the polymicrobial infection.

3. Methicillin-susceptible *S. aureus* (MSSA) false negative in case 31.

The JI Panel failed to detect MSSA, which was later confirmed through synovial fluid culture as part of a chronic polymicrobial PJI (involving MSSA and *Streptococcus oralis*). After the correct diagnosis, targeted antibiotics were prescribed for both pathogens.

(B) Culture false negative.

MSSA false negative in case 8.

In a polymicrobial infection involving MSSA and *Escherichia coli*, the JI Panel detected both pathogens, whereas synovial fluid culture identified *E. coli* alone. This case illustrates the complementary pathogen detection of the two methods. Despite the false-negative culture result, targeted antibiotics were prescribed for both pathogens, thereby ensuring appropriate treatment.

(C) JI Panel false positive.

MRSA false positive in case 44.

The JI Panel incorrectly identified MRSA, but the patient was ultimately classified as having a non-infectious condition. This highlights the potential for false-positive results in the JI Panel, even for MRSA, and emphasizes the importance of combining clinical assessment, culture-based results, and multidisciplinary input to reach a definitive diagnosis, especially when the diagnostic result is discordant with clinical presentation.

## DISCUSSION

This prospective study compared the diagnostic performance of the JI Panel and synovial fluid culture in patients with suspected PJI of the knee. Key findings suggest that while the JI Panel is a valuable tool for acute PJI diagnosis and enhancing antibiotic stewardship, its role in chronic PJI diagnosis and situations involving recent antibiotic exposure may be limited.

Identification of the pathogen is essential for optimal treatment in PJI. In the current study, the sensitivity of the JI Panel for diagnosing all PJI cases was low (50.0%), but its performance was higher when focusing on the on-JI Panel pathogens (86.4%). Both the JI Panel and the on-JI Panel demonstrated high sensitivity for detecting pathogens in acute infections, with 80.0% and 94.1%, respectively. This highlights the JI Panel’s strong performance in diagnosing acute PJI, likely due to the presence of *S. aureus,* which is more easily identified by the Panel. For chronic PJI, both the JI Panel and the on-JI Panel showed low sensitivity (16.7% and 60.0%, respectively). This was likely due to the predominance of coagulase-negative staphylococci (e.g., *S. epidermidis*) in chronic infections, which the JI Panel struggles to detect, affecting its diagnostic accuracy for these cases. These pathogens are often detected in low bacterial loads and embedded in mature biofilm. In addition, they are slow-growing or fastidious, making them difficult to detect using both molecular and culture-based approaches. Furthermore, the exclusion of these organisms from the current JI Panel configuration significantly limits its utility in chronic PJI.

The JI Panel is an effective diagnostic tool for NJI, demonstrating high sensitivity in detecting associated pathogens (12–14, 18, 25). However, the role of the JI Panel in diagnosing PJI remains less clear due to differences in the causative pathogens involved in NJI compared to PJI (26). A summary of JI Panel studies in different types of joint infections provides important insights (Table 7). A study involving 107 cases of acute PJI showed that the JI Panel demonstrated a diagnostic agreement of 95.3% for these patients, like its performance in NJI (12). However, Schoenmakers et al. stratified data based on joint type, revealing varying sensitivities: 83% for NJI, 73% for late acute PJI, but 30% for early acute PJI (18). The study concluded that while the JI Panel offers clear benefits for NJI and late acute PJI, its performance is limited in early acute PJI due to the exclusion of *S. epidermidis*, a pathogen commonly associated with early infections. Berinson et al. highlighted the limitations of the JI Panel in diagnosing PJI, specifically pointing out that the Panel does not cover important pathogens such as coagulase-negative staphylococci and *C. acnes* (14). Azad et al. found that the JI Panel’s performance for diagnosing PJI was less accurate because many important pathogens, including *S. epidermidis* and *C. acnes*, are not included in the kit (13). Though no data on acute or chronic PJI were provided, *S. epidermidis* and *C. acnes* are more commonly associated with chronic infections (12). Therefore, it is likely that the study predominantly involved chronic PJI (13). In the current study, the high diagnostic performance of the JI Panel for acute PJI can be explained by the distribution of pathogens involved. On-JI Panel pathogens accounted for 85% (17/20) of the cases, while off-JI Panel pathogens represented only 15% (3/20). This distribution aligns with well-established microbiological patterns of acute PJI, where high-virulence organisms such as *S. aureus* and *S. agalactiae* predominate. By contrast, chronic PJI is often caused by low-virulence organisms such as *S. epidermidis* and *C. acnes*, which are associated with biofilm formation and more indolent clinical courses.

**TABLE 7:** Summary of JI Panel studies in different joint infection types^*a*^

Study (authors, year of report)	Study type	Location	Case no.	NJI	PJI (acute/chronic)	Other joint infections	Sensitivity/PPA (On-JI Panel)	Specificity/NPA
Moran et al. (16)	Retrospective (frozen), prospective (fresh)	USA	167 (frozen 63, fresh 104)	Frozen 48, fresh 87	Frozen 14, fresh 17	Frozen 1	Frozen: 92.8% Fresh: 71.4% (not detailed NJI or PJI)	Frozen: 97.1% Fresh: 94.8%
Esteban et al. (15)	Retrospective, multicenter	USA, Canada, France	1544	850	442	252 (Containing information unknown)	Overall, 90.5% NJI 88.2% PJI 92.0% (not detailed acute or chronic)	Overall, 99.6% NJI 99.6% PJI 99.4%
Berinson et al. (14)	Retrospective, single-center	USA	123	17	18	0	100% (Not detailed acute or chronic)	100%
Azad et al. (13)	Retrospective, single-center	USA	60	0	60	0	91% (not detailed acute or chronic)	100%
Gaillard et al. (17)	Prospective, multicenter	French	307	176	131	0	84.9% (overall agreement) (not detailed NJI or PJI)	100% (overall agreement)
Hoffman et al. (25)	Prospective, single-center	Israel	57	57	0	0	92%	100%
Schoenmakers et al (18)	Prospective, single-center	Netherlands	45	12	33 (19/14)	0	NJI: 83.3% Early acute PJI: 30.0% Late acute PJI: 73.3%	NJI: 100% Early acute PJI: 100% Late acute PJI: 100%
Salar-Vidal et al. (19)	Prospective, multi-center	Spain, Portugal	262	132	105	25 (Osteosynthesis material)	69% agreement	91.9%
Gardete-Hartmann et al. (21)	Retrospective, single-center	Austria	268	0	141 (early acute 31, late acute 26, chronic 89)	0	41.4% (overall) Early acute PJI: 65% Late acute PJI: 89.4% Chronic PJI: 55.6%	91.1% (overall) Early acute PJI: 10% Late acute PJI: 28.5% Chronic PJI: 12.9%
Pascual et al. (20)	Retrospective, multi-center	Europe, Middle East	1527	873	398	256 (unknown)	85.7% (overall agreement)	Not calculated
Saeed et al. (12)	Retrospective, multi-center	UK Ireland	399	292	107	0	91.6% (overall agreement)	93% (overall agreement)
Lin et al. (current study)	Prospective, single-center	Taiwan	54	0	43 (20/23)	0	86.4%	90.9%

^*a*^ NJI, native joint infection; PJI, prosthetic joint infection; PPA, positive percent agreement; NPA, negative percent agreement.

The finding that only 40% of JI Panel results were positive in chronic PJI compared to 63.5% of conventional culture underscores a key limitation of the JI Panel for diagnosing chronic PJI (21). The limited diagnostic performance of the JI Panel in chronic PJI is a key observation in the current study. The fact that off-JI Panel pathogens (such as coagulase-negative staphylococci and *C. acnes*) accounted for a large proportion of the pathogens (72.2%) in chronic infections explains the poor sensitivity (16.7%) observed in detecting chronic PJI. This is significantly lower than the 100% sensitivity of synovial fluid culture. The predominance of coagulase-negative staphylococci in our chronic PJI cohort is consistent with previous reports that identified *S. epidermidis* as a leading pathogen in chronic PJI cases (18, 27). This microbial profile underscores a critical limitation of the present JI Panel, which lacks coverage of several key pathogens commonly implicated in chronic infections. The high proportion of off-JI Panel pathogens in our chronic PJI cases further highlights the importance of preserving traditional culture methods, which provide broader pathogen detection and remain the gold standard in diagnosing indolent or biofilm-associated infections (15).

The impact of prior antibiotic exposure on the diagnostic yield of synovial fluid culture in PJI is a significant challenge, as it can lead to culture-negative results (28), where antibiotic exposure can decrease the number of viable pathogens available for detection, especially when the organism is already suppressed or eradicated by previous antibiotics (29). In the current study, despite the shared impact with prior antibiotic exposure, the on-JI Panel performed comparably to synovial fluid culture, making it a valuable diagnostic tool in these settings, particularly for acute PJI. Moreover, the molecular approach may provide a more consistent and rapid diagnosis, especially in cases where the pathogen is not readily cultivable or present in low quantities. While abstaining from antibiotics for at least 2 weeks is recommended for optimal culture results (30), this is not always feasible in real-world clinical settings, particularly in acutely infected patients. In these cases, the on-JI Panel could help bridge the gap in diagnosis when culture results are inconclusive or delayed.

The on-JI Panel enables early pathogen identification and rapid administration of targeted antibiotics within 18.2 hours, significantly improving the timeliness of treatment, especially in acute PJI. This is much faster than the 84.6 hours typically required when relying solely on culture-based methods to detect off-JI Panel pathogens. This reduced reliance on empiric broad-spectrum antibiotics helps mitigate the side effects associated with their prolonged use, such as antibiotic resistance, diarrhea, and other secondary infections (31). The on-JI Panel stands out as a game changer in the management of acute PJI, where delays in diagnosis and treatment can lead to serious complications such as septic shock, bone destruction, or prosthesis failure (32). The ability to move directly from empiric antibiotics to targeted therapy is a key advantage.

Discrepancy results between the JI Panel and conventional culture are an important issue in the diagnostic landscape of PJI (19). While the JI Panel offers rapid and sensitive detection, the discrepancy rate has been reported up to 7% of cases (16), which is similar to the 11.1% (6/54) observed in the present study. The sample quality and handling can also contribute to discrepancies. For example, the JI Panel typically uses a smaller volume of fluid for analysis, which may limit the detection of pathogens present in low quantities. In addition, the rapid processing time of the JI Panel can sometimes miss slow-growing or fastidious pathogens that may require a longer culture period (16, 33). By contrast, synovial fluid culture involves more time-intensive processes, allowing for broader microbial growth, but is also prone to contamination, false negatives, or loss of pathogens due to the presence of antibiotics or inappropriate handling (15). In cases of discrepancy in results, further confirmatory methods, such as PCR assays and next-generation sequencing (NGS), have been employed to resolve uncertainty and ensure accurate pathogen identification (13). Both the JI Panel and synovial fluid culture can yield false-positive or false-negative results; thus, intraoperative tissue culture remains the most important method for the confirmation of pathogens, consequently guiding decisions on whether to proceed with empiric antibiotics, initiate targeted therapy, or consider additional tests. A multidisciplinary approach involving orthopedic surgeons, infectious disease specialists, and microbiologists is crucial to determine the most appropriate treatment plan for PJI cases (20).

Our study has several limitations. First, it has a small sample size, and it is a single-center design. The second limitation was the absence of additional molecular confirmatory tests, such as NGS, for the discrepant results, which may leave some diagnostic uncertainties unresolved. Due to limitations in the quantity of the IUO version of the JI Panel, we were unable to perform repeated JI Panel testing to resolve discrepancies, which could have provided a more definitive diagnostic conclusion (16). Third, our study focused solely on PJI of the knee, and it remains uncertain whether the findings can be directly applied to other prosthetic joint types, such as those in the hip or shoulder (34, 35). Different joint types may harbor distinct pathogen profiles, which could influence the diagnostic performance of the JI Panel and conventional cultures. Fourth, while this study provides important insights into the diagnostic accuracy of the JI Panel for acute and chronic PJI of the knee, general conclusions about its performance across all species and resistance markers cannot be drawn. The results are limited to the specific pathogens and resistance profiles tested in our cohort. Lastly, this study did not evaluate long-term clinical outcomes such as infection resolution or reinfection rates. Although we recognize the importance of outcome-based assessment, almost all patients in our cohort have not yet reached a sufficient follow-up duration due to the prospective nature of our study design. The recently proposed Delphi-based international consensus criteria for PJI define eradication as a minimum of 24 months without clinical or laboratory evidence of infection (36). To avoid premature conclusions, outcome data were not included at this stage. Future follow-up studies are planned to assess the real-world clinical efficacy of rapid, diagnostic-guided treatment strategies.

Despite these limitations, the prospective design of the study, combined with fresh synovial fluid samples collected by a single senior orthopedic surgeon, lends significant credibility to the findings.

In summary, the JI Panel demonstrated high sensitivity for pathogen detection and significantly reduced the time to initiate targeted antibiotic therapy in acute PJI of the knee. We recommended the use of the JI Panel in acute PJI of the knee for its role in rapid pathogen detection and enhanced antibiotic stewardship. Further studies are encouraged to validate these findings in larger, multicenter cohorts and to explore their applicability in chronic PJI and other joint types.
